# Childhood Maltreatment and Revictimization in Young Adulthood: Is Problematic Substance Use a Mediator? A Linked Survey–Register Data Study

**DOI:** 10.1177/08862605241301787

**Published:** 2024-11-28

**Authors:** Lars Roar Frøyland, Kristian Heggebø

**Affiliations:** 1 NOVA – Norwegian Social Research, OsloMet – Oslo Metropolitan University, Norway

**Keywords:** childhood maltreatment, violence and abuse, alcohol consumption, drug disorders, coping strategies, mediation analysis, longitudinal study, administrative register data, retrospective survey information

## Abstract

Victims of childhood maltreatment often experience revictimization later in life. However, there is scant knowledge of the explanatory mechanisms that generate this phenomenon. Problematic substance use is a maladaptive coping strategy that may increase the risk of revictimization after maltreatment. We used linear regressions and mediation analyses to investigate the explanatory role of problematic substance use in the association between childhood maltreatment and violent revictimization in young adulthood. We analyzed linked survey-register data on a sample of senior high school graduates in Norway (*n* = 3,156) who were followed longitudinally until ages 32 to 33 (2021). Ordinary least squares regression analyses showed that childhood maltreatment was associated with both problematic substance use and violent revictimization, adjusted for a wide range of covariates. Moreover, problematic substance use was associated with an increased risk of violent revictimization in young adulthood. Mediation analyses showed that about one eighth (12.0%) of the association between childhood maltreatment and violent revictimization was mediated by problematic substance use. Sensitivity tests indicated that the mediation may be susceptible to unmeasured confounding. Thus, it is unclear whether the mediating role of problematic substance use can be interpreted causally within a counterfactual framework. In conclusion, reducing problematic substance use among victims of childhood maltreatment may mitigate the risk of violent revictimization later in life. Early detection and tailored treatment of problematic substance use could be one way to prevent violent revictimization following childhood maltreatment. Therefore, equitable access to primary and specialized healthcare services is essential, both for victims of maltreatment and individuals with habits of (borderline) problematic substance use. However, the majority of the maltreatment-revictimization pathway (88.0%) is not statistically explained by substance use, which implies that other mechanisms are also at play.

Childhood maltreatment is a public health problem that affects millions of children globally (Moody et al., 2018; Stoltenborgh et al., 2015). Maltreatment can take the form of physical, sexual, or emotional abuse, as well as neglect through failure to meet basic needs. It can lead to severe short- and long-term health consequences, such as physical injuries, neurocognitive deficits, psychological dysfunctions, and even death (Carr et al., 2020; Gilbert et al., 2009). Childhood maltreatment can also have substantial negative impacts on adult socioeconomic outcomes, including unemployment, financial strain, poverty, and social-benefits receipt (Bunting et al., 2018; Herbert et al., 2023). Furthermore, maltreatment is associated with an increased risk of revictimization later in life (Fereidooni et al., 2024; Walker & Wamser-Nanney, 2023). Thus, childhood maltreatment potentially sets off a chain reaction of disadvantages that may accumulate and deepen over the life course. Hence, knowledge about ways to interrupt this chain reaction is necessary.

Maltreatment is usually a traumatic life experience that needs to be dealt with. Individual differences in coping strategies may explain why some victims of traumatic events fare better while others fare worse. Coping refers to the controlled and effortful stress–response strategies used to regulate emotion, cognition, behavior, physiology, and the environment when exposed to traumatic incidents (Compas et al., 2017); these strategies are different from more subconscious adaptive responses, sometimes labeled defense mechanisms. Avoidance coping strategies, where the victim avoids stressors or their own stress responses, have been linked to maltreatment. One way to avoid stress responses is to self-medicate through substance use, which has been described as a potentially maladaptive coping strategy after traumatic experiences (Savage & Crowley, 2018).

The association between childhood maltreatment and revictimization is firmly established in the literature (Fereidooni et al., 2024; Goemans et al., 2023; Li et al., 2019; Walker & Wamser-Nanney, 2023), but there is scarce knowledge regarding the explanatory mechanisms involved. As highlighted in two recent reviews (Fereidooni et al., 2024; Walker & Wamser-Nanney, 2023), substance misuse could be an important mechanism. Childhood maltreatment may instigate problematic substance use in adolescence, which puts individuals in settings where the risk of violent victimization is high.

This study analyzed linked survey-register data. Retrospective accounts of childhood maltreatment (before age 13) were collected in a survey administered to senior high school graduates aged 18 to 19 in Norway (*n* = 3,156). Childhood maltreatment was defined as experiencing parental violence, witnessing partner violence, and/or sexual abuse prior to the age of 13. Prospective information on violent victimization (up to the ages 32–33) was derived from an administrative register consisting of police crime records. Violent victimization was defined as being registered as a victim of violent crimes (e.g., physical assault, bodily harm, violent threats). The linked data material was used to examine the association between childhood maltreatment and revictimization in young adulthood. The mediating role of one potential explanatory mechanism—problematic substance use in adolescence—was also investigated. Problematic substance use was defined as lifetime use of cannabis or other narcotics, alcohol intoxication more than once a week the previous year, and/or being registered with substance use-related criminal charges during adolescence.

## Previous Research

There is consistent and strong evidence of the importance of childhood maltreatment for later-life revictimization risk. The existing literature on sexual trauma is especially well developed. In a meta-analytic review, Walker et al. (2019) concluded that close to half of all children exposed to childhood sexual abuse reported future sexual revictimization. Associations have also been established between childhood sexual abuse and physical victimization in adulthood (Desai et al., 2002). The existing literature on physical revictimization is more limited, though. A survey-based study from the United States found significantly higher violent victimization among adults with documented cases of childhood maltreatment, such as neglect, physical victimization, and sexual abuse, compared to a matched control group (Widom et al., 2008). Another study of the same data material found elevated rates of physical crime victimization 10 to 15 years later (McIntyre & Widom, 2011). Review studies have established associations between childhood maltreatment and intimate partner violence victimization in adulthood (Li et al., 2019) as well as peer victimization in adolescence (Goemans et al., 2023).

Most of the maltreatment-revictimization literature originates in the United States and other high-income countries (Walker & Wamser-Nanney, 2023), and there is little research on at-risk subgroups, such as indigenous people and LGBTQ+ individuals, often because of data limitations (e.g., small survey samples).

## Explanatory Mechanism

According to the self-medication hypothesis (Khantzian, 1997), some victims of traumatic experiences may use substances to cope with negative feelings and/or memories related to previous trauma, including childhood maltreatment. This maladaptive coping strategy can lead to problematic substance use, especially if sustained over a long period. Substance use in general (Boles & Miotto, 2003) and alcohol and drug disorders in particular (Seid et al., 2022) are associated with violent victimization. Problematic substance use may lead to types of behaviors that make it difficult to protect oneself, both with regard to intoxication levels and the likelihood of ending up in dangerous situations (Walker & Wamser-Nanney, 2023).

Even though the maltreatment-revictimization and maltreatment-substance use pathways are firmly established in the literature, the potential mediating role of substance use in the association between childhood maltreatment and revictimization is unclear. A survey-based U.S. study found that physical maltreatment in childhood led to a greater risk of alcohol- or drug-abuse diagnosis during early adulthood, which was associated with higher criminal victimization 10 to 15 years later (McIntyre & Widom, 2011). However, the impact of physical maltreatment during childhood on crime victimization in adulthood was not mediated by alcohol- or drug-abuse diagnosis. In contrast, another survey-based study from the United States found that heavy drinking mediated the association between childhood maltreatment and revictimization in adolescence (12- to 17-year-olds), but not in later developmental periods (Smith et al., 2018). Studies have also investigated the mediating role of substance use in the association between sexual abuse and sexual revictimization. A common conclusion is that both sexual abuse and substance use are associated with sexual revictimization, but the indirect pathway is insignificant (Culatta et al., 2020; Fargo, 2009; Messman-Moore & Long, 2002).

The present study adds to the literature by examining the childhood maltreatment-revictimization association using linked survey-register data in a new context—the Norwegian welfare state.

## Research Context

Norway is a comprehensive welfare state, meaning that the public authorities (the state, counties, and municipalities) provide key services and income protection to citizens throughout the life course. Education, including high school and higher education, is offered free of charge or is heavily subsidized. There are a few out-of-pocket payments in the country’s universal healthcare system, and various support services and treatment regimes, for both maltreatment trauma and substance misuse, are, in theory, readily available. However, there are important barriers to access due to the complex and multilayered health system, which comprises numerous healthcare providers.

Compared to other parts of the world, the prevalence of childhood maltreatment is low in Nordic welfare states (Kloppen et al., 2015). Rates of violent crime and crime in general are also comparatively low (Lappi-Seppälä & Tonry, 2011). Thus, the risks of childhood maltreatment and violent victimization are probably lower in the present research context than in most other countries.

## Empirical Expectations

According to Smith et al. (2018), there is a need for more research on childhood maltreatment, substance use, and revictimization across different developmental periods. The current study addresses this need by examining whether problematic substance use in adolescence mediates the relationship between childhood maltreatment and violent victimization in young adulthood. The empirical findings may therefore reveal to what extent problematic substance use is an explanatory mechanism through which childhood maltreatment affects violent victimization. Based on previous research and theoretical reasoning concerning the self-medication hypothesis, we expected the following three results: (1) childhood maltreatment is associated with both problematic substance use and more violent victimization in young adulthood; (2) problematic substance use is associated with more violent victimization in young adulthood; and (3) part of the association between childhood maltreatment and revictimization is mediated through problematic substance use.

## Methods

### Procedures and Participants

The data material consists of survey information from the Youth Violence study (*n* = 6,468; 58.3% females) conducted among Norwegian senior high school graduates aged 18 to 19 years, linked with information from administrative registers up to 2021 (ages 32–33 years). The survey was conducted in 2007 in 67 schools that comprised a nationally representative sample of senior high schools in Norway. The participating pupils mainly attended academic programs that prepared them for enrollment in higher education (87.6%). The study was approved by the Norwegian Data Protection Authority (ref. no. 06/01512) and the Regional Committees for Medical and Health Research Ethics, South East (ref. no. 2017/526).

All the pupils in the sampled schools were invited to participate in the survey. The analytical sample was restricted to 18- to 19-year-olds who consented to register data linkages (*n* = 3,156; 61.0% female; 48.8% of the initial sample). The participants who consented to register data linkages were more often female and had less often migration backgrounds (Frøyland & Andersen, 2023). Thus, the analytical sample was slightly skewed, which might have led to some bias.

### Measures and Variables

#### Outcome Measure

*Violent victimization* in young adulthood was derived from police registers (crime records). Those registered as victims of a violent crime after the age of 18 were coded 1 (else = 0). Violent crimes were defined as acts represented in chapters 24, 25, and 27 of the Norwegian Penal Code (e.g., physical assault, bodily harm, homicide, violent threats, robbery, and extortion).

#### Independent Variable

*Childhood maltreatment* was self-reported by the respondents at age 18–19, covering experiences with severe parental violence, witnessing severe partner violence, and sexual abuse before the age of 13. Four items, inspired by the Parent–Child Conflict Tactics Scale (Straus, 1979; Straus et al., 1998), assessed physical violence from the respondent’s father or mother (e.g., “hit you with a fist” and “beat you up”). The same number of items evaluated witnessing partner violence toward one’s mother or father. Six items measured sexual abuse (vaginal penetration, anal penetration, oral sex, other forms of sex, attempted rape, and rape). Those who reported at least one instance of parental violence, witnessing partner violence, or sexual abuse prior to the age of 13 were coded 1 (else = 0).

#### Mediator Variable

*Problematic substance use* combined self-reported information and police crime records; the aim here was to separate respondents with potentially harmful substance use during adolescence from others. The self-reported information included lifetime use of cannabis, other narcotics, and prescription drugs to get intoxicated, as well as drinking to intoxication several times a week during the previous 12 months at age 18. Police crime records include registered criminal charges at ages 13 to 18 for drug-related crimes and alcohol-related disorderly conduct. Those who reported drug use or alcohol intoxication more than once a week, as well as those who had been registered with substance use-related criminal charges during adolescence, were coded 1 (else = 0).

#### Covariates

A range of covariates were included in the statistical analyses. Five covariates were based on the administrative registers. Females were coded 1 on registered *gender* at birth (male = 0). Respondents with two foreign-born parents were coded 1 on *immigrant background* (else = 0). Respondents living with a single parent at age 16 were coded 1 on *single-parent household* (else = 0). *Parental education level* when the respondent was 16 years old (a proxy for social background) was based on the parent with the highest education level. Dummy variables for “no education after junior high school” (yes = 1), “1–4 years of education after junior high school” (yes = 1), and “5–8 years of education after junior high school” (yes = 1) were included, with “8+ years of education after junior high school” as the reference category. *School grades* from junior high school were included as proxies for cognitive abilities (range 21–65). Two covariates were based on the survey data. Respondents who had *lived in public housing* were coded 1 (else = 0). *Parental substance use problems* were assessed using two items regarding whether the respondent’s father or mother had, or had had, alcohol- or substance-use problems. Those who responded “to a substantial degree” or “yes, clearly” were coded 1 (else = 0).

### Statistical Analyses

The linked survey-register data were examined with descriptive statistics, linear regression, and mediation analysis. We started with descriptive statistics split by childhood maltreatment prior to age 13 (yes/no). Then, a correlation matrix was utilized to inspect for multicollinearity issues. Correlations between two continuous variables were calculated as Pearson’s *r*, between continuous and binary variables as point biserial *r*, between two binary variables as the phi (*r*_φ_) coefficient, and between categorical and other variables as Spearman’s rank correlation coefficient. We proceeded with ordinary least squares (OLS) linear regression analyses, with and without a range of covariates included in the model specifications. Finally, a mediation analysis was conducted to explore how the independent variable (childhood maltreatment) affected the outcome measure (violent revictimization) through the mediator variable (problematic substance use).

The aim of the mediation analysis was to shed empirical light on problematic substance use as an explanatory mechanism. The term “explanatory” is used here in a statistical sense, that is, how much of the maltreatment-revictimization pathway that is statistically explained by the studied mechanism (problematic substance use). Whether this (potential) explanatory mechanism can be interpreted causally is debatable, however. Various approaches to causal inference exist, and the counterfactual framework is the perhaps most common one (VanderWeele, 2015). The *temporal order* of the included variables is considered crucial in all understandings of causality. In the present study, the mediation model used data on maltreatment in childhood (before age 13), problematic substance use in adolescence (ages 13–18), and violent victimization after age 18 to establish a clear temporal order. However, in mediation analyses, causal inference also hinges on a strong assumption of a lack of confounding in either of the pathways of the mediation model (Valente et al., 2017). Various risk factors, negative life events, and persisting disadvantages may co-occur with childhood maltreatment and substance use patterns, and thereby pose a challenge for the interpretation of results. We adjusted for a wide range of covariates to reduce such omitted variable bias, but residual confounding seems likely. Reverse causation is a less pressing issue in this study due to the clear temporal order. However, information on childhood maltreatment was collected retrospectively, and it is possible that problematic substance use leads to over- or underreporting of unfortunate childhood experiences. Thus, caution is needed when interpreting the empirical findings causally within a counterfactual framework.

In a counterfactual framework, mediation analyses return three main effects: the total effect, the natural direct effect (NDE), and the natural indirect effect (NIE). NDEs are changes in the outcome measure caused by changes in the independent variable while holding the mediator constant, while NIEs are changes in the outcome measure caused by changes in the mediator while holding the independent variable constant. The mediation analyses were conducted using the mediation package (Tingley et al., 2014) in R-4.4.1 (R Core Team, 2023). The mediation model can be expressed by the following two linear equations:

(1) $${\mathrm{viovic}}_{i}=\beta_{0}+\beta_{1}{\mathrm{subst}}_{i}+\beta_{2}{\mathrm{maltreat}}_{i}+\beta_{3}C_{i}+\in_{yi},$$

(2) $${\mathrm{subst}}_{i}=\gamma_{0}+\gamma_{1}{\mathrm{maltreat}}_{i}+\gamma_{2}C_{i}+\in_{yi},$$

where viovic indicates violent victimization in young adulthood; subst represents problematic substance use; maltreat stands for childhood maltreatment; C is the vector of covariates, and ∈ indicates the residuals.

Controlling for relevant covariates, particularly those affecting the pathway between the mediator (problematic substance use) and the outcome (violent revictimization), is important when conducting mediation analyses. Inspired by Smith et al. (2018), we included a range of individual and familial covariates, such as gender, school grades, and parental substance use problems. Continuous covariates were centered at their grand mean. Bias-corrected and accelerated confidence intervals were estimated using bootstrap sampling with 10,000 replications, as recommended by Tingley et al. (2014).

In models with interaction terms, there were no significant variations in the indirect paths of problematic substance use on violent victimization according to maltreatment status (*P* = . 458). The moderated mediation models showed insignificant gender differences both in the direct (*P* = .976) and indirect effects (*P* = .964). Therefore, we opted for parsimonious model specifications without interaction terms and gender moderation.

**Figure 1.**
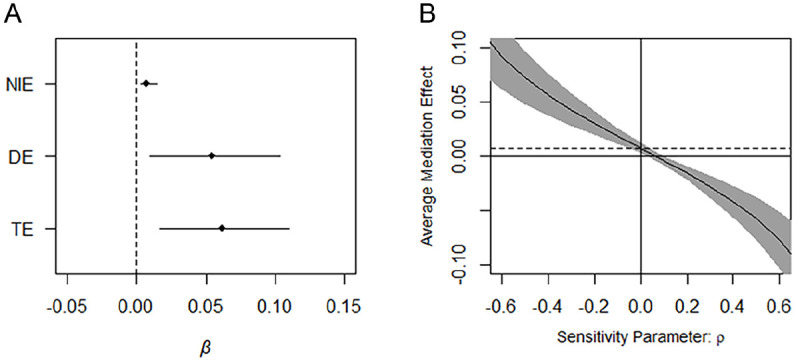
(A) Counterfactual mediation analysis and (B) sensitivity analysis. *Note*. NIE = natural indirect effect; DE = direct effect; TE = total effect.

We also conducted a sensitivity test with the medsens function of R’s mediation package (Tingley et al., 2014). A hypothetical parameter (*p*) that correlated with the mediator and the outcome was included in the analyses. The sensitivity test consisted of varying the strength of the hypothetical correlation to investigate how the estimated indirect effect changed and whether the coefficient was reduced to zero at some point. The results are presented in Figure 1B, which shows the indirect effect at different values of *p*.

Missing data were handled with listwise deletion. The missing data for the included variables were very low (1.4% max). The mediation analyses carried out with multiple imputed data did not differ from the empirical findings obtained using listwise deletion. The code and data for reproducing the statistical analyses are available at https://osf.io/m64rc. The AGReMA guidelines for how to communicate the findings of mediation analyses based on observational studies were followed (Lee et al., 2021).

## Results

### Descriptive Statistics and Linear Regressions

Table 1 presents the descriptive statistics for all the included variables split by exposure to childhood maltreatment prior to age 13. Among those who reported no maltreatment (*n* = 2,900), 8.1% had been violently victimized after the age of 18. The rate was roughly double (16.1%) among those who reported childhood maltreatment (*n* = 255). Problematic substance use was also significantly higher among those who experienced childhood maltreatment: 34.5% versus 16.8%. The respondents who reported childhood maltreatment more often were female, had immigrant backgrounds, had parents with shorter education, lived in single-parent households, lived in public housing, reported parental substance use problems, and had poorer school grades, compared to those who did not report maltreatment. Table 2 shows that the correlations between the included variables were mostly weak (Cohen, 1988). The strongest correlations were for school grades-parental education (*r* = .30) and public housing-immigrant background (*r* = .20). Hence, multicollinearity was probably not a major issue in the analyses.

**Table 1.** Sample Characteristics.

	No Childhood Maltreatment (*n* = 2,900)	Childhood Maltreatment (*n* = 255)	*P* Value of Group Difference
Variables	% (*n*)	% (*n*)	
Outcome			
Violent victimization	8.1 (234)	16.1 (41)	<.001***
Mediator			
Problematic substance use	16.8 (486)	34.5 (88)	<.001***
Covariates			
Female	60.0 (1,740)	72.9 (186)	<.001***
Immigrant background	3.4 (100)	13.7 (35)	<.001***
Single-parent household	18.1 (526)	35.3 (90)	<.001***
Parental education			
Junior high school	4.2 (123)	12.7 (32)	<.001***
1–4 years after junior high school	39.9 (1,157)	44.2 (111)	.204
5–8 years after junior high school	39.0 (1,132)	31.5 (79)	.022*
8+ years after junior high school	16.8 (487)	11.6 (29)	.039*
Lived in public housing	6.1 (174)	14.0 (35)	<.001***
Parental substance use problems	3.8 (110)	15.3 (39)	<.001***
School grades (21–65), *M* (*SD*)	49.6 (6.24)	47.0 (6.97)	<.001***

Note. *M* = mean; *SD* = standard deviation. *P < .05. **P  < .01. ***P  < .001.

**Table 2.** Correlation Matrix for All the Included Variables.

Variables	1.	2.	3.	4.	5.	6.	7.	8.	9.	10.
1. Childhood maltreatment	—	.08	.13	.07	.14	.12	−.09	.09	.15	−.11
2. Violent victimization	.08	—	.09	−.06	.02	.05	−.06	.03	.04	−.11
3. Problematic substance use	.13	.09	—	.01	−.04	.11	.01	.01	.09	−.16
4. Female	.07	−.06	.01	—	−.01	.05	−.09	.01	.06	.09
5. Immigrant background	.14	.02	−.04	−.01	—	−.03	−.05	.20	.03	−.10
6. Single-parent household	.12	.05	.11	.05	−.03	—	−.07	.01	.12	−.11
7. Parental education level	−.09	−.06	.01	−.09	−.05	−.07	—	−.02	−.05	.30
8. Lived in public housing	.09	.03	.01	.01	.20	.01	−.02	—	.09	−.11
9. Parental substance use problems	.15	.04	.09	.06	.03	.12	−.05	.09	—	−.07
10. School grades	−.11	−.11	−16	.09	−.10	−.11	.30	−.11	−.07	—

*Note*. Correlations of |.04| and above are significantly different from zero at *P* < .05. Correlations between two continuous variables were calculated as Pearson’s *r*, between scaled and binary variables as point biserial *r*, between two binary variables as the phi (*r*_φ_) coefficient, and between categorical and other variables as Spearman’s rank correlation coefficient.

Table 3 shows the results of the stepwise OLS linear regression models in which childhood maltreatment, problematic substance use, and a range of covariates were included with violent victimization in young adulthood as the outcome measure. Model 1 shows that childhood maltreatment (*b* = 0.08) was significantly related to violent victimization when included as the only independent variable. In Model 2, problematic substance use was added, which was also significantly associated with violent victimization (*b* = 0.06); the coefficient for childhood maltreatment slightly declined (*b* = 0.07). The third and final model specification included a range of covariates. The coefficients for childhood maltreatment (*b* = 0.05) and problematic substance use (*b* = 0.05) declined somewhat, but they remained statistically significant. Table 4 presents the results of the linear regression models with problematic substance use as the outcome measure. Model 1 shows that childhood maltreatment was significantly (*b* = 0.18) related to problematic substance use. The association diminished slightly (*b* = 0.15) when the covariates were added in Model 2.

**Table 3.** Linear Regression Analysis with Violent Victimization in Young Adulthood as the Dependent Variable.

Variable	Model 1		Model 2		Model 3	
	*b* [95% CI]	*P Value*	*b* [95% CI]	P Value	*b* [95% CI]	P Value
(Intercept)	0.08 [0.07, 0.09]	<.001***	0.07 [0.06, 0.08]	<.001***	0.06 [0.03, 0.09]	<.001***
Childhood maltreatment	0.08 [0.04, 0.12]	<.001***	0.07 [0.03, 0.11]	<.001***	0.05 [0.02, 0.09]	.005**
Problematic substance use			0.06 [0.03, 0.09]	<.001***	0.05 [0.02, 0.08]	<.001***
Female					−0.04 [−0.06, −0.02]	<.001***
Immigrant background					0.01 [−0.04, 0.06]	.720
Single-parent household					0.02 [0.00, 0.05]	.088
Parental education level (ref. 8+ years after junior high school)						
Junior high school					0.01 [−0.04, 0.07]	.586
1–4 years after junior high school					0.05 [0.02, 0.07]	.003**
5–8 years after junior high school					0.04 [0.01, 0.07]	.011*
Lived in public housing					0.03 [−0.02, 0.07]	.226
Parental substance use problems					0.02 [−0.02, 0.07]	.342
School grades					0.00 [−0.01, 0.00]	<.001***
Adjusted *r*^2^	.006		.012		.026	

*Note.* 95% CI = 95% confidence interval of *b*. *P < .05. **P < .01. ***P < .001.

**Table 4.** Linear Regression Analysis with Problematic Substance Use as the Dependent Variable.

Variable	Model 1		Model 2	
	*b* [95% CI]	*P* Value	*b* [95% CI]	*P* Value
(Intercept)	0.17 [0.15, 0.18]	<.001***	0.21 [0.17, 0.25]	<.001***
Childhood maltreatment	0.18 [0.13, 0.23]	<.001***	0.15 [0.10, 0.20]	<.001***
Female			0.01 [−0.02, 0.03]	0.690
Immigrant background			−0.11 [−0.18, −0.04]	.001**
Single-parent household			0.07 [0.04, 0.10]	<.001***
Parental education level (ref. 8+ years after junior high school)				
Junior high school			−0.11 [−0.18, −0.04]	.003**
1–4 years after junior high school			−0.07 [−0.11, −0.03]	<.001***
5–8 years after junior high school			−0.05 [−0.09, −0.01]	.009**
Lived in public housing			−0.01 [−0.07, 0.04]	.714
Parental substance use problems			0.11 [0.05, 0.18]	<.001***
School grades			−0.01 [−0.01, −0.01]	<.001***
Adjusted *r*^2^	.015		.054	

*Note*. 95% CI = 95% confidence interval of *b*. * *P* < .05. **P < .01. ***P < .001.

### Mediation Analysis

Figure 1A presents the findings of the mediation analysis. The coefficient for the total effect was .062 (95% CI [.017, .111]; *P* = .008), which indicates that the probability of violent victimization in young adulthood would have increased on average by 6.2 percentage points if all the participants had been exposed to childhood maltreatment. The coefficient was reduced to .054 (95% CI [.009, .104]; *P* = .020) if problematic substance use was held constant at the level of the participants not exposed to maltreatment (the direct effect). Finally, the mediation analysis revealed a significant indirect effect of childhood maltreatment on violent victimization via problematic substance use. The probability of revictimization in young adulthood would have been reduced by 0.7 percentage points if problematic substance use had been reduced to the level of those not exposed to childhood maltreatment (*b* = .007; 95% CI [.003, .014]; *P* < .001). Also, 12.0% of the total effect of childhood maltreatment on violent victimization was mediated through problematic substance use. All the estimates were adjusted for a range of covariates, such as gender, immigrant background, childhood living conditions, parental substance use problems, and school grades.

Figure 1B presents the results of the sensitivity test. The mediating role of problematic substance use in the association between childhood maltreatment and violent victimization was apparently susceptible to unmeasured residual confounding. If a vector of unmeasured confounders positively correlated with the residuals of the mediator and the outcome measure at .07 or higher, the indirect effect would be reduced to zero. Thus, caution is needed when interpreting the mediation identified in this study.

We conducted several additional analyses to check the robustness of the mediation analysis. When the outcome measure was restricted to being registered as a victim of violent crime after the age of 25, the indirect effect was noticeably reduced to *b* = .003 (95% CI [.000, .008]; *P* = .072) and statistically significant on the 90% level. Restricting violent crime victimization to only include probable “street crimes” (physical assault, bodily harm, robbery and extortion, and violence from a public officer) reduced the indirect effect to insignificance (*P* = .132). Removing criminal charges for substance use from the mediator variable did not change the results, nor did changing the explanatory variable to include only sexual abuse. Changing the explanatory variable to parental violence only, however, reduced the indirect effect to insignificance (*P* = .792). Thus, the indirect effect was insignificant in certain model specifications, but the mediation analysis was quite robust overall.

## Discussion

Childhood maltreatment is a global public health problem with potentially long-term harmful impacts on a broad array of domains. Maltreatment early in life may set off a chain reaction of negative events and disadvantages that accumulate and deepen over the life course. Hence, knowledge about ways to interrupt this chain reaction is needed. To contribute to this end, the present study investigated the explanatory role of problematic substance use in the association between childhood maltreatment and violent victimization in young adulthood. Linked survey-register data that allowed following a sample of senior high school graduates (*n* = 3,156) in Norway longitudinally until ages 32 to 33 (year 2021) was analyzed with OLS linear regression models and mediation analyses. We tested the following three empirical expectations:

1. Childhood maltreatment is associated with problematic substance use and more violent victimization in young adulthood.
2. Problematic substance use is associated with more violent victimization in young adulthood.
3. Part of the association between childhood maltreatment and revictimization is mediated through problematic substance use.

The results show that childhood maltreatment significantly increased the risk of both problematic substance use and revictimization in young adulthood, which confirms previous studies (McIntyre & Widom, 2011; Widom et al., 2008). There were significantly higher levels of violent revictimization among those reporting problematic substance use, again confirming previous studies (Boles & Miotto, 2003; Seid et al., 2022). Furthermore, we found that part (12.0%) of the association between childhood maltreatment and revictimization was mediated via problematic substance use, which is in line with Smith et al. (2018) but contradicts McIntyre and Widom (2011). Problematic substance use therefore appears to partly explain, in a statistical sense, the maltreatment-revictimization pathway. It is unclear, though, whether this suggested explanatory mechanism can be interpreted causally. Numerous relevant covariates were included in the mediation analysis, which should reduce omitted variable bias. On the other hand, the mediating role of problematic substance use seemed to be susceptible to unmeasured confounding (Figure 1B). Furthermore, reverse causation is still a concern since information on childhood maltreatment and substance use habits was collected simultaneously. Thus, it is uncertain whether the mediation can be interpreted causally within a counterfactual framework, although the statistical relationship was quite strong.

Children that experience maltreatment are likely exposed to other co-occuring hardships. Various risk factors (e.g., emotional distress, harsh parenting), negative life events (e.g., family death, parental job loss), and persisting disadvantages (e.g., bullying, household poverty) could lead to revictimization, either directly or through problematic substance use (Walker & Wamser-Nanney, 2023). Moreover, childhood maltreatment (and/or problematic substance use) could lead to other negative life outcomes than revictimization. Examples include not completing education, labor market marginalization, social benefit utilization, union dissolution, and health deterioration (Bunting et al., 2018; Herbert et al., 2023).

Overall, this study indicates that problematic substance use may be an explanatory mechanism in the association between childhood maltreatment and later-life revictimization. Problematic substance use can be targeted through intervention efforts (Smith et al., 2018). Therefore, early detection of problematic substance use, together with tailored support and treatment, could be used to prevent violent victimization, and possibly other disadvantages, following childhood maltreatment. Consequently, ensuring equitable access to primary and specialized healthcare services is essential, both for victims of maltreatment and individuals with habits of (borderline) problematic substance use.

A rather small share of the association between childhood maltreatment and violent revictimization (about one eighth, 12.0%) was mediated by problematic substance use. Future research should investigate other explanatory mechanisms that may be of importance for the maltreatment-revictimization pathway. Review studies have highlighted posttraumatic stress and other psychological symptoms, risky sexual behavior, emotion dysregulation, and dissociation as potential mechanisms (Fereidooni et al., 2024; Walker & Wamser-Nanney, 2023). Most of these suggested mechanisms point to how childhood maltreatment can affect psychological functioning of victims, which in turn may increase the risk of revictimization. An alternative pathway is how childhood maltreatment may impact social relations and relationships, thereby facilitating revictimization (Fereidooni et al., 2024). Studies with long follow-ups that enable detailed analyses of life courses from childhood to adulthood and into old age are particularly in want. Furthermore, studies conducted in other countries than the United States and Norway may shed new light on the topic.

### Strengths and Limitations

This study adds to the existing literature by examining linked survey-register data. Senior high school graduates were surveyed retrospectively concerning childhood maltreatment, and they were followed prospectively regarding violent victimization using crime records. To the best of our knowledge, this is the first study on this topic not originating in the United States. Methodologically, mediation analysis was employed with measures of the independent variable (maltreatment), the mediator (problematic substance use), and the outcome (violent victimization) during separate stages of life (childhood, adolescence, and young adulthood).

Several limitations should also be acknowledged. First, information on childhood maltreatment was collected retrospectively at ages 18 to 19, which makes the measure susceptible to recall bias. Second, all self-reported information was collected at the same time, which might affect the temporal order of the included variables. For instance, high school graduates with problematic substance use could tend to over- or underreport difficult childhood experiences. Third, whether and to what extent the participants actually had substance use *problems* was unclear. Fourth, the establishment of causal inference within a counterfactual framework was challenging due to the observational nature of the data. For instance, the assumption of no unmeasured confounding in either of the pathways of the mediation analysis may have been violated. Fifth, the participants were senior high school graduates, which implies a somewhat selected sample with regard to sociodemographic characteristics. Sixth, those not present at school and those who declined to participate may have differed nonnegligibly in terms of unobservable characteristics. Seventh, those who consented to register data linkages differed from the initial survey sample on gender and migration background, which might have slightly biased the estimates. Eighth, individuals with certain characteristics (e.g., low socioeconomic status) may be more prone to being registered as victims of violent victimization by the police, which could have led to some bias in the outcome measure. Ninth, the external validity of this study may be limited due to peculiarities of the Norwegian research context. Tenth, the study did not examine at-risk subgroups, such as indigenous people and LGBTQ+ individuals; hence, it did not increase diversity in the existing literature.

### Conclusion

This study investigated the explanatory role of problematic substance use in the association between childhood maltreatment and violent victimization in young adulthood. Linked survey-register data, which allowed to follow senior high school graduates in Norway longitudinally, was analyzed with linear regression models and mediation analysis. The findings indicate that problematic substance use was a mediator in the pathway from childhood maltreatment to violent revictimization. However, it is unclear whether the mediating role of problematic substance use can be interpreted causally within a counterfactual framework. About one eighth (12.0%) of the association was mediated by problematic substance use in adolescence. Thus, other mechanisms are likely to be also of importance.
